# Long-term outcome and prognosis of mixed histiocytosis (Erdheim-Chester disease and Langerhans Cell Histiocytosis)

**DOI:** 10.1016/j.eclinm.2024.102658

**Published:** 2024-05-27

**Authors:** Francesco Pegoraro, Matthias Papo, Fleur Cohen-Aubart, Francesco Peyronel, Gianmarco Lugli, Irene Trambusti, Gildas Baulier, Mathilde de Menthon, Tanguy Le Scornet, Eric Oziol, Nicole Ferreira-Maldent, Olivier Hermine, Benoit Faucher, Dirk Koschel, Nicole Straetmans, Noémie Abisror, Benjamin Terrier, François Lifermann, Jerome Razanamahery, Yves Allenbach, Jeremy Keraen, Sophie Bulifon, Baptiste Hervier, Annamaria Buccoliero, Frederic Charlotte, Quentin Monzani, Samia Boussouar, Natalia Shor, Annalisa Tondo, Stephane Barete, Ahmed Idbaih, Abdellatif Tazi, Elena Sieni, Zahir Amoura, Jean-François Emile, Augusto Vaglio, Julien Haroche

**Affiliations:** a Sorbonne University, Internal Medicine Department 2, Institut E3M, French Reference Centre for Histiocytosis, Pitié-Salpȇtrière Hospital, CIMI INSERM-UMRS 1135, Assistance Publique-Hôpitaux de Paris, Paris, France; b Hematology and Oncology Department, Meyer Children’s Hospital IRCCS, Florence, Italy; c Department of Experimental and Clinical Medicine, University of Florence, Florence, Italy; d Nephrology and Dialysis Unit, Meyer Children’s Hospital IRCCS, Florence, Italy; e Rare Disease Center, Meyer Children’s Hospital IRCCS, Florence, Italy; f Department of Clinical and Experimental Medicine, University of Pisa, Pisa, Italy; g Internal Medicine and Clinical Immunology Department, Périgueux University Hospital, Périgueux, France; h Paris-Saclay University, Internal Medicine and Clinical Immunology Department, Bicetre Hospital, Assistance Publique - Hôpitaux de Paris, Le Kremlin Bicêtre, France; i Internal Medicine Department, Hotel Dieu, Nantes University Hospital, Nantes, France; j Internal Medicine Department, Béziers Hospital, Béziers, France; k Internal Medicine Department, University Hospital of Tours, Tours, France; l Hematology Department, Hôpital Necker - Enfants Malades, Assistance Publique-Hôpitaux de Paris, Paris, France; m Internal Medicine Department, La Timone Hospital, Assistance Publique-Hôpitaux de Marseille, Marseille, France; n Internal Medicine and Pneumology Department, Fachkrankenhaus Coswig, Lung Center, Coswig and Medical Department I, Division of Pneumology, University Hospital Carl Gustav Carus, TU Dresden, Dresden, Germany; o Hematology Department, University Hospital Saint-Luc, Brussels, Belgium; p Internal Medicine Department, Saint Antoine Hospital, Assistance Publique-Hôpitaux de Paris, Paris, France; q Internal Medicine Department, Referral Center for Rare Autoimmune and Systemic Diseases, Cochin Hospital, Assistance Publique-Hôpitaux de Paris, Paris, France; r Internal Medicine Department, Dax University Hospital, Dax, France; s Internal Medicine and Clinical Immunology Department, Dijon University Hospital, Dijon, France; t Internal Medicine Department, Sorbonne University, INSERM UMRS 974, Pitié-Salpêtrière Hospital, Assistance Publique-Hôpitaux de Paris, Paris, France; u Internal Medicine and Immunology Department, Cornouaille Hospital Center, Quimper, France; v Paris-Saclay University, Faculty of Medicine, Le Kremlin-Bicêtre, France; w INSERM UMR_S 999, Le Kremlin-Bicêtre, France; x Respiratory and Intensive Care Medicine Department, Pulmonary Hypertension National Referral Center, Bicêtre Hospital, Assistance Publique-Hôpitaux de Paris, Le Kremlin-Bicêtre, France; y Internal Medicine Department, Saint Louis Hospital, Assistance Publique-Hôpitaux de Paris, Paris, France; z Pathology Department, Meyer Children’s Hospital IRCCS, Florence, Italy; aa Pathology Department, Pitié-Salpêtrière Hospital, Sorbonne University, Assistance Publique-Hôpitaux de Paris, Paris, France; ab Polyvalent and Oncologic Radiology Department–Musculoskeletal Unit, Pitié-Salpêtrière Hospital, Paris, France; ac Cardiovascular and Thoracic Imaging Unit, Pitié-Salpêtrière Hospital, Assistance Publique-Hôpitaux de Paris, Institute of Cardiometabolism and Nutrition (ICAN), Paris, France; ad Neuro-Radiology Department, Pitié-Salpêtrière Hospital, Assistance Publique-Hôpitaux de Paris, Paris, France; ae Sorbonne University, Dermatology Unit, Pitié-Salpêtrière Hospital, Assistance Publique-Hôpitaux de Paris, Paris, France; af Sorbonne University, Neuro-Oncology Department, Paris Brain Institute - ICM, Inserm, CNRS, Pitié-Salpêtrière Hospital, DMU Neurosciences, Assistance Publique-Hôpitaux de Paris, Paris, France; ag UFR de Médecine, Paris Cité University, Paris, France; ah INSERM UMR 976 Saint Louis Research Institute, Paris, France; ai Pneumology Department, Saint Louis Hospital, Assistance Publique-Hôpitaux de Paris, Paris, France; aj EA4340 BECCOH, Versailles SQY University, Pathology Department, Ambroise Paré Hospital, Assistance Publique-Hôpitaux de Paris, Boulogne, France; ak Department of Biomedical, Experimental and Clinical Sciences “Mario Serio”, University of Florence, Florence, Italy

**Keywords:** Erdheim-Chester disease, Langerhans, BRAF, Histiocytosis, Mixed histiocytosis, Pediatric histiocytosis

## Abstract

**Background:**

Erdheim-Chester disease (ECD) is a rare histiocytosis that may overlap with Langerhans Cell Histiocytosis (LCH). This “mixed” entity is poorly characterized. We here investigated the clinical phenotype, outcome, and prognostic factors of a large cohort of patients with mixed ECD-LCH.

**Methods:**

This retrospective study was performed at two referral centers in France and Italy (Pitié-Salpêtrière Hospital, Paris; Meyer Children’s Hospital, Florence). We included children and adults with ECD diagnosed in 2000–2022 who had biopsy-proven LCH, available data on clinical presentation, treatment and outcome, and a minimum follow-up of one year. Outcomes included differences in clinical presentation and survival between mixed ECD-LCH and isolated ECD; we also investigated response to treatments and predictors of survival in the mixed cohort. Survival was analyzed using the Kaplan-Maier method and differences in survival with the long-rank test. Cox regression models were used to evaluate the potential impact of age and gender on survival and to identify predictors of non-response and survival.

**Findings:**

Out of a cohort of 502 ECD patients, 69 (14%) had mixed ECD-LCH. Compared to isolated ECD, mixed ECD-LCH occurred more frequently in females (51 *vs.* 26%, *p* < 0.001) and in patients with multisystem disease (≥4 sites). Mixed ECD-LCH more frequently involved long bones (91 *vs.* 79%, *p* = 0.014), central nervous system (51 *vs.* 34%, *p* = 0.007), facial/orbit (52 *vs.* 38%, *p* = 0.031), lungs (43 *vs.* 28%, *p* = 0.009), hypothalamic/pituitary axis (51 *vs.* 26%, *p* < 0.001), skin (61 *vs.* 29%, *p* < 0.001), and lymph nodes (15 *vs.* 7%, *p* = 0.028); the *BRAF*^*V600E*^ mutation was also more frequent in mixed ECD-LCH (81 *vs.* 59%, *p* < 0.001). Targeted treatments (BRAF and/or MEK inhibitors) induced response more frequently than conventional therapies (interferon-α, chemotherapy), either as first-line (77 *vs.* 29%, *p* < 0.001) or as any line (75 *vs.* 24%, *p* < 0.001). After a median follow-up of 71 months, 24 patients (35%) died. Survival probability was comparable between ECD alone and mixed ECD-LCH (log-rank *p* = 0.948). At multivariable analysis, age at diagnosis (HR 1.052, 95% CI 1.008–1.096), associated hematologic conditions (HR 3.030, 95% CI 1.040–8.827), and treatment failure (HR 9.736, 95% CI 2.919–32.481) were associated with an increased risk of death, while lytic bone lesions with a lower risk (HR 0.116, 95% CI 0.031–0.432).

**Interpretation:**

Mixed ECD-LCH is a multisystem disease driven by the *BRAF*^*V600E*^ mutation and targeted treatments are effective. Age at diagnosis, bone lesion patterns, associated hematologic conditions, and treatment failure are the main predictors of death in mixed ECD-LCH.

**Funding:**

None.

**Research in context**

**Evidence before this study**

The overlap between Erdheim-Chester disease (ECD) and Langerhans Cell Histiocytosis (LCH) is reported in 10–15% of ECD patients. Given the paucity of available data, the clinical phenotype and outcome of this mixed entity remain poorly characterized.

**Added value of this study**

Mixed ECD-LCH was found in 14% of a cohort of 502 ECD patients. The ECD and LCH diagnoses were metachronous in half patients. Compared to patients with isolated ECD, patients with mixed ECD-LCH more frequently had multisystem involvement and BRAF mutations. Novel targeted treatments (BRAF and MEK inhibitors) appeared more effective than conventional agents (interferon-α, chemotherapy). No significant difference in survival was detected between ECD and mixed ECD-LCH. Age at diagnosis, associated hematologic conditions, and treatment failure were strong predictors of death in mixed ECD-LCH, while lytic bone lesions were associated with prolonged survival.

**Implications of all the available evidence**

The mixed ECD-LCH phenotype is rare but needs to be carefully searched for, also because of its frequent multisystem involvement and the favorable response to targeted treatments. The clinical presentation and the type of involved organs have a strong prognostic value that can guide clinical management.

## Introduction

Langerhans Cell Histiocytosis (LCH) and Erdheim-Chester Disease (ECD) are the two main forms of histiocytosis.^1^^,^^2^ ECD is a rare, systemic histiocytic disorder that mainly occurs in adults, characterized by lesions rich in foamy CD68+ CD1a-histiocytes and fibrosis.^3^ LCH is a typical disease of childhood but can occur in adults as well, and is caused by tissue infiltration by CD1a and langerin-staining histiocytes.^4^ Both diseases are thought to have a dual clonal-inflammatory nature: mutations in genes involved in the MAPK pathway such as *BRAF*^*V600E*^ are detected in both conditions, but fibro-inflammatory mechanisms also play a role. Moreover, germline genetic variants^5^^,^^6^ and environmental risk factors^7^ confer susceptibility to the disease.

An overlap between ECD and LCH is reported in around 10–15% of adults^8^^,^^9^ and 40% of children with ECD.^10^ In the largest series reported to date, which included 23 patients with mixed ECD-LCH, the phenotype of patients with mixed ECD-LCH was more similar to that of isolated ECD, compared to isolated LCH, and most patients harbored the *BRAF*^*V600E*^ mutation. These patients were diagnosed before the targeted therapy era, and only half of them responded to conventional treatments.^11^ Therefore, the best therapeutic approach to mixed ECD-LCH is unclear. In particular, the efficacy of targeted therapies (*e.g.*, BRAF inhibitors, BRAFi; MEK inhibitors, MEKi), which have revolutionized patient management and improved outcomes of ECD in the last decade,^12^, ^13^, ^14^, ^15^ remains to be established in mixed ECD-LCH.

In the present work, performed on a large cohort of patients with ECD followed at two referral centers for histiocytic disorders in France and Italy, we were able to collect 69 cases of mixed ECD-LCH. Herein, we report their clinical, histopathological, and molecular features, analyze response to treatment and long-term survival, and compare their phenotype and outcome with those of patients with ECD alone.

## Methods

### Study design

This is a retrospective observational study performed at two referral centers in France and Italy (Pitié-Salpêtrière Hospital, Paris; Meyer Children’s Hospital IRCCS, Florence). Patients diagnosed from 2000 to 2022 were included in the study if they met the eligibility criteria.

### Patients

We screened pediatric and adult patients with ECD or mixed ECD-LCH, defined according to the latest Consensus on ECD,^16^ and followed at the two study centers. Patients were included when they fulfilled the following criteria: a) biopsy-proven diagnosis of LCH b) biopsy-proven diagnosis of ECD or clinical diagnosis of ECD according to stringent criteria as stated in the last ECD Consensus^16^; c) available data on clinical presentation, treatment, and outcome, with a minimum follow-up of one year.

### Procedures

Clinical and laboratory data included demographics, signs and symptoms at presentation, organ involvement at diagnosis, pathology and molecular studies, treatment regimens and responses, survival, and causes of death.

Organ involvement was defined according to the latest consensus recommendations for ECD.^16^ The assessment included: bone involvement, either sclerotic or lytic^17^^,^^18^; central nervous system (CNS) involvement by parenchymal lesions, meningeal lesions, and neurodegenerative disease^19^; hypothalamic–pituitary abnormalities resulting in central diabetes insipidus and/or enlargement, infiltration, or abnormal brightness of pituitary gland at MRI scans^16^; facial–orbital involvement consisting of infiltrating masses causing exophthalmos, diplopia, visual impairment, and/or craniofacial bone involvement^20^; cardiac involvement (*i.e.*, pericardial infiltration, myocardial infiltration causing right atrioventricular pseudotumor, infiltration of the right atrioventricular sulcus, or right coronary artery sheathing at MRI)^21^^,^^22^; circumferential soft tissue sheathing of the large vessels^23^; involvement of the lungs (interlobular septal thickening, ground glass opacities, centrilobular opacities, pleural thickening/effusions; or middle and upper lobe cysts/nodules with interstitial thickening)^24^; infiltration of peri-renal fat (“hairy kidney”), ureters, and peri-adrenal space, hydroureteronephrosis, kidney atrophy^9^; xanthelasma-like lesions of the periorbital space and non-specific patches and papulonodular lesions^25^; and other less frequently involved sites (*e.g.*, lymph nodes, gastrointestinal system). Data on associated hematologic conditions (*e.g.*, myeloproliferative neoplasms, MPN, and myelodysplastic syndrome, MDS) were collected as well.^26^

The pathologic diagnosis of ECD was made by referral national pathologists, based on the latest classification of the Histiocyte Society.^1^ All patients included in the study also had a pathologic diagnosis of LCH.^1^ The *BRAF* mutational status was assessed on diagnostic biopsies by pyrosequencing, next-generation sequencing (NGS), or digital droplet PCR. Where available, the NGS panel included other genes of the MAPK pathway (*e.g.*, *MAP2K1*, *KRAS*, and *NRAS*).

### Outcomes

The study outcomes included the identification of the differences in clinical presentation between mixed ECD-LCH and ECD alone, the assessment of response to different treatments in patients with mixed ECD-LCH, and the definition of predictors of treatment failure (*i.e.*, lack of response in three or more lines of treatment) and death.

The response to treatment was assessed through medical chart review and was defined as a composite end-point, combining clinical, radiologic,^27^ and metabolic^28^ response criteria, as previously reported,^15^ and was categorized as “response”, “stable disease (SD)” or “progressive disease (PD)”. “Response” included at least one of the following: a) the complete resolution of clinical manifestations of active disease with stable radiologic or metabolic studies; b) a diameter reduction of at least 30% of the target lesions on radiologic studies without the appearance of new lesions; or c) complete metabolic response (resolution of 18 F-fluorodeoxyglucose (18-FDG) uptake at 18-FDG positron emission tomography, PET). PD was defined either as the development of new histiocytosis-related manifestations, a lesion diameter increase of at least 20%, the appearance of new lesions on radiologic studies, an increase of at least 30% in target lesion activity, or the appearance of new lesions on PET scans. SD was defined as neither response nor PD.

### Statistical analysis

Categorical variables were reported as numbers and percentages, whereas continuous variables as median and interquartile range (IQR). Comparisons between groups were made using the chi-square or Fisher’s exact tests for categorical variables, and the Mann–Whitney test for continuous variables, after assessing normality using the D’Agostino-Pearson and Kolmogorov–Smirnov tests. Correlation analyses were performed by computing Spearman’s correlation coefficients. Predictors of treatment failure in the ECD-LCH group were investigated through univariable and multivariable Cox regression models. ORs are expressed by exp(B) values and are reported with their respective 95% confidence intervals (95% CIs). The incidence of death throughout follow-up was estimated using a Kaplan–Meier survival analysis. The log-rank test was used to compare survival between groups. A Cox regression model was used to investigate the potential confounding effect of age and sex on survival. We also compared the clinical phenotype and survival of mixed ECD-LCH with those of a cohort of 138 patients with isolated ECD, matched for age and gender. For matching, we used a 1:2 greedy nearest neighbor matching algorithm without replacement. Predictors of death were identified through univariable and multivariable Cox regression models. HRs are expressed by exp(B) values and are reported with their 95% CIs. In the multivariate Cox regression model, variables were entered using a forward selection method, after checking for multicollinearity. A two-sided *p*-value ≤ 0.05 was considered to indicate statistical significance. Statistical analyses were performed using IBM SPSS Statistics for Windows (version 25.0).

### Ethics statement

The study was approved by the Institutional Review Board of the Meyer Children’s Hospital IRCCS (Florence, Italy) and the Ethical Board CESREES #2814848 (NCT04437381). Patients provided written informed consent for the study.

### Role of the funding source

There was no funding for this study. All authors had access to the study dataset and agreed on the decision to submit for publication.

## Results

Five hundred and two patients with ECD, diagnosed between 2000 and 2022 and followed at the two participating centers, were screened. Of them, 69 (14%) had a biopsy-proven diagnosis of LCH and were therefore included in the study; nine patients had clinical features of LCH (mainly lytic bone lesions) but lacked pathology confirmation and were excluded.

### Clinical presentation

The baseline characteristics of the 69 patients with mixed ECD-LCH are shown in Table 1 and in Supplementary Fig. S1. Of them, 35 (51%) were female; the median age at symptom onset was 50 years (IQR 36–62), while the median age at LCH and ECD diagnoses was 52 (IQR 36–65) and 55 (IQR 44–67) years, respectively. The most frequent symptomatic clinical features at presentation included bone pain (n = 21), diabetes insipidus (n = 16), skin lesions (n = 9), and respiratory symptoms (n = 7).

**Table 1:** Differences in baseline clinical characteristics between patients with mixed ECD-LCH and a large cohort of patients with ECD alone followed at the two study institutions.

	Mixed ECD-LCH (N = 69)	ECD alone (N = 424)	*p*-value
Age at LCH diagnosis, years	52 (36–65)	NA	NA
Age at ECD diagnosis, years	55 (44–67)	60 (50–69)	**0.040**
Female sex	35 (51%)	109 (26%)	**<0.001**
Organ involvement			
Long bone	63 (91%)	334 (79%)	**0.014**
Sclerotic	56 (81%)	334 (79%)	0.651
Lytic	41 (59%)	NA	NA
CNS	35 (51%)	144 (34%)	**0.007**
Neurodegenerative disease	21 (30%)	78 (18%)	**0.021**
Facial/orbit	36 (52%)	163 (38%)	**0.031**
Heart	27 (39%)	177 (42%)	0.682
Large vessel	42 (61%)	238 (56%)	0.461
Lung	30 (43%)	119 (28%)	**0.009**
Interstitial/pleural	21 (30%)	119 (28%)	NA
Upper lobes, cystic	9 (13%)	NA	NA
Retroperitoneal	43 (62%)	298 (70%)	0.184
Hypothalamic/Pituitary	35 (51%)	111 (26%)	**<0.001**
Skin	42 (61%)	123 (29%)	**<0.001**
Xanthelasma-like	22 (32%)	NA	NA
Papules/patches	27 (39%)	NA	NA
Lymph nodes	10 (15%)	29 (7%)	**0.028**
GI	9 (13%)	40 (9%)	0.352
Associated hematologic neoplasms^b^	11 (16%)	66 (16%)	0.936
Number of involved sites	6 (4–7)	4 (3–6)	**<0.001**
≥4 involved sites	56 (81%)	275 (65%)	**0.001**
Somatic mutations^a^			
*BRAF*^*V600E*^	54/67 (81%)	227/387 (59%)	**<0.001**
*MAP2K1*	5/67 (7%)	36/387 (9%)	0.627
No mutations	8/67 (12%)	116/387 (30%)	**0.002**
Other mutation	0 (0%)	8/387 (2%)	NA

Data are n (%), median (IQR), or n/N (%). Statistically significant *p*-values are reported in bold.

ECD, Erdheim-Chester disease; LCH, Langerhans Cell Histiocytosis; NA, not applicable; CNS, central nervous system; GI, gastrointestinal.

a Data not available for all patients.

b Including myeloproliferative neoplasm, myelodysplastic syndromes, and non-Hodgkin lymphoma.

Almost all patients underwent a complete assessment at diagnosis (*e.g.*, PET-CT in 93%; chest CT in 86%, and brain MRI in 94%). Imaging and metabolic studies revealed sclerotic, bilateral bone involvement in 56 patients (81%) and lytic bone lesions in 41 (59%), while 34 (49%) had both sclerotic and lytic lesions. Thirty-six patients (52%) had facial/orbital involvement, and 35 (51%) CNS abnormalities (62% of them had neurodegenerative disease). Thirty-five patients (51%) had hypothalamic and/or pituitary infiltration, and 42 (61%) had skin lesions (22 xanthelasma-like and 27 had erythematous papules/inflammatory patches). Involvement of the peri-renal space was found in 43 patients (62%), while peri-aortic and cardiac infiltration in 42 (61%) and 27 (39%), respectively. Thirty patients (43%) had lung involvement, including interlobular septal thickening, ground-glass or centrilobular opacities (*i.e.*, ECD-like findings) in 21 (30%) and cysts or nodules of the upper lobes (*i.e.*, LCH-like findings) in nine (13%). Finally, 11 patients (16%) had associated hematologic conditions (MDS in five patients, chronic myelomonocytic leukemia in three, non-Hodgkin lymphoma in two, and myelofibrosis in one).

### Histological and molecular findings

LCH biopsy sites mostly included the bone (n = 33) and the skin (n = 24), whereas most ECD lesions were diagnosed through the analysis of biopsies of the pathologic peri-renal tissue (n = 24) (Supplementary Table S1). In 37 patients, the ECD and LCH diagnoses were synchronous, while in 27 of the remaining 32 patients, the ECD diagnosis was made after that of LCH, with a median delay of four years (IQR 2–14). Finally, in five patients, the diagnosis of ECD preceded that of LCH.

**Fig. 1:**
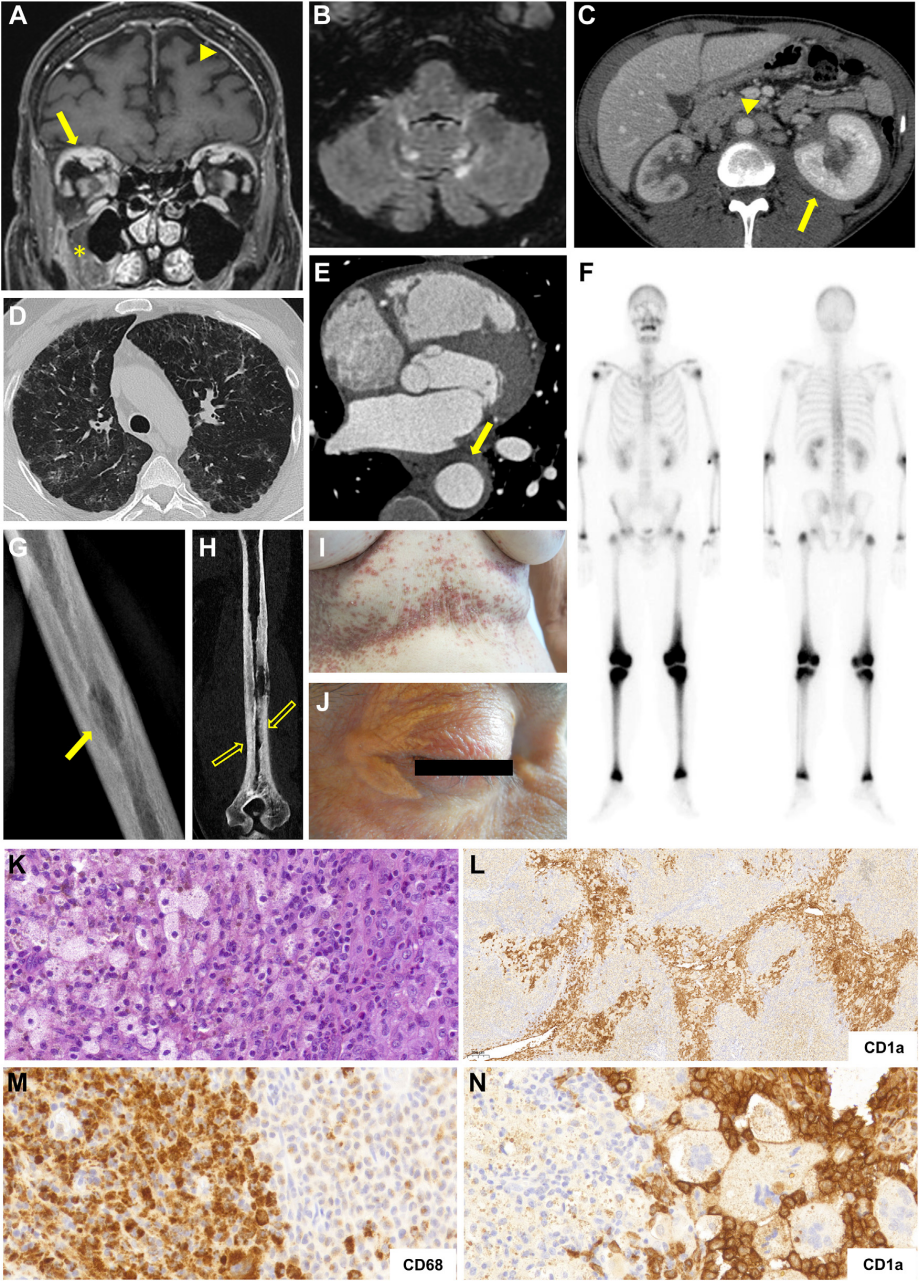
Typical imaging and pathology findings in mixed ECD-LCH. **A**. Brain MRI gadolinium-enhanced T1 FAT SAT reveals orbital fat histiocytic infiltration (arrows), pachymeningeal involvement (arrowhead), and right premaxillary involvement (asterisk). **B**. Brain MRI T2 FLAIR sequence showing degenerative changes with symmetrically increased signal intensity within the cerebellar parenchyma. **C**. Axial abdominal contrast-enhanced CT scan showing periaortic and bilateral perinephric infiltrates (“hairy kidneys”, arrow). **D**. Axial thin-section CT scan of the lungs showing multiple cysts and nodules in the upper lobes. **E**. Axial arterial contrast-enhanced CT scan showing circumferential periaortic infiltration (arrow). **F**. 99 mTC bone scan demonstrating multiple areas of abnormally increased uptake including a symmetric uptake in the femurs, tibia, knees, and ankles. Other locations in the appendicular skeleton show mild uptake. Left: anterior view; right: posterior view. **G, H**. Humeral X-ray (G) and CT-scan (H) showing a lytic lesion with periosteal reaction (arrow); osteosclerosis and cortical thickening are present on the lower half of the humeral diaphysis (empty arrows). **I**. Brown and purplish papules with crusted lesions in the breast folds and upper abdomen. **J**. Xanthelasma-like lesions of the eyelids. **K**. H&E staining of mixed ECD-LCH bone involvement, showing large histiocytes with abundant clear cytoplasm and smaller histiocytes with eosinophilic cytoplasm (200×) **L, N**. Low (L, 40×), and high (N, 400×) magnification of the same biopsy showing infiltration by CD1a + LCH cells. **M**. The same sample stained with CD68+ showing a strong positivity of the foamy ECD cells (200×).

Fifty-four out of 67 tested patients (81%) harbored the *BRAF*^*V600E*^ mutation. Twelve of the remaining 13 were tested by NGS for other mutations of the MAPK pathway, and five had a *MAP2K1* mutation (Supplementary Fig. S1). A panel depicting clinical manifestations and pathology findings in our patients is shown in Fig. 1.

### Differences in baseline features between mixed ECD-LCH and ECD alone

The clinical characteristics of the ECD-LCH patients were compared with those of a cohort of 424 patients with ECD alone. Patients with mixed ECD-LCH were diagnosed at a younger age (55 *vs.* 60 years, *p* = 0.040), were more likely to be female (51 *vs.* 26%, *p* < 0.001) and to present with multisystem disease, since the median number of involved sites was 6 *vs.* 4 (*p* < 0.001) and the proportion of patients with ≥4 involved sites was 81 *vs.* 65% (*p* = 0.001). Indeed, they more frequently had involvement of long bones (91 *vs.* 79%, *p* = 0.014), CNS (51 *vs.* 34%, *p* = 0.007, including neurodegeneration, 30 *vs.* 18%, *p* = 0.021) facial/orbital structures (52 *vs.* 38%, *p* = 0.031), lung (43 *vs.* 28%, *p* = 0.009), hypothalamic/pituitary (51 *vs.* 26%, *p* < 0.001), skin (61 *vs.* 29%, *p* < 0.001), and lymph nodes (15 *vs.* 7%, *p* = 0.028). Also, a greater proportion of patients with mixed ECD-LCH harbored a *BRAF*^*V600E*^ mutation (81% *vs.* 59%, *p* < 0.001) (Table 1). Most of these phenotypic differences were confirmed in an age- and gender-matched comparison between mixed forms and ECD alone (except for long bone, facial-orbit, and lymph node involvement, which did not reach statistical significance in this latter analysis) (Supplementary Table S2).

### Response to treatment and predictors of response

First-line therapies included chemotherapy regimens in 17 patients (25%), interferon-α in 25 (36%), and targeted treatments (*i.e.*, BRAFi and/or MEKi) in 22 (32%); other molecules, such as anti-IL1 and mammalian target of rapamycin (mTOR) inhibitors were less frequently used (Supplementary Table S3). Forty-one patients (59%) required more than one line of treatment because of disease progression or drug-related toxicity: in most cases, second- or third-line therapy consisted of BRAFi and/or MEKi (35 out of 59 therapeutic instances, 61%), or anti-cytokine drugs (n = 10) (Supplementary Table S3). Considering all 126 therapeutic instances, the best objective responses were observed in patients receiving BRAFi and/or MEKi (Supplementary Table S3). As compared to conventional treatments (*i.e.*, interferon-α, anti-cytokine, chemotherapy), BRAFi and/or MEKi were more likely to induce a response either as first-line (77 *vs.* 29%, *p* < 0.001) or as any line of treatment (75 *vs.* 24%, *p* < 0.001). The same response profile was confirmed for targeted treatments *vs.* interferon-α (first line, 77% *vs.* 32%, *p* = 0.003; any line, 75% *vs.* 31%, *p* < 0.001).

At univariable logistic regression analysis, the use of BRAFi and/or MEKi was negatively associated with treatment failure (OR 0.308, 95% CI 0.095–0.996; *p* = 0.049), similar to sclerotic bone lesions (OR 0.265, 95% CI 0.073–0.962; *p* = 0.043) and the presence of the *BRAF*^*V600E*^ mutation (OR 0.239, 95% CI 0.069–0.829; *p* = 0.024). However, no significant predictors were found at the multivariable analysis (Supplementary Table S4).

### Survival analysis and prognostic factors

After a median follow-up of 71 months (IQR 35–116), 24 patients (35%) died. Causes of death were disease-related in 13 patients (54%) and mostly consisted of CNS and lung involvement; the remaining deaths were attributable to cancer in most cases (n = 6).

**Fig. 2:**
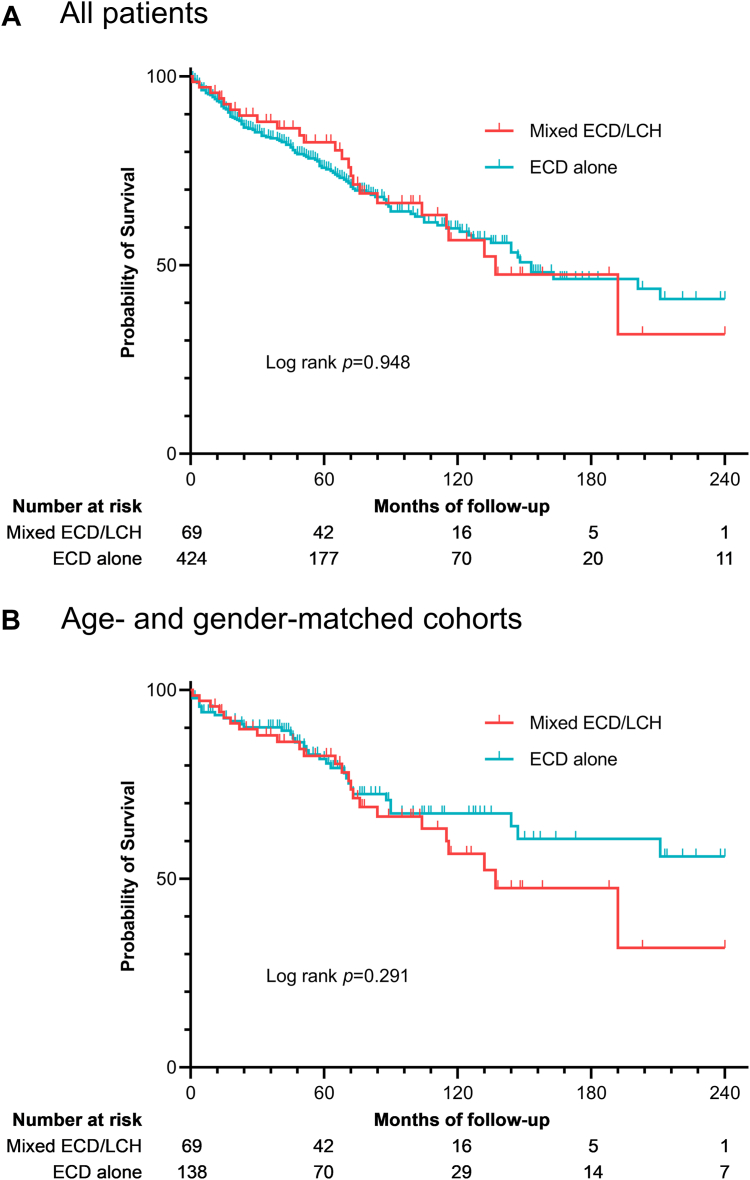
Kaplan–Meier estimates of survival of patients with mixed ECD-LCH *vs.* patients with ECD alone. **A**. Mixed ECD-LCH *vs.* the whole cohort of isolated ECD (424 patients); **B**. Mixed ECD-LCH *vs.* an age- and gender-matched cohort of 138 patients with isolated ECD (1:2 ratio). *Abbreviations used in the figure*: ECD, Erdheim-Chester Disease; LCH, Langerhans Cell Histiocytosis.

No significant differences in terms of survival were found between patients with mixed ECD-LCH and patients with ECD alone (log-rank *p* = 0.948; Fig. 2), even after applying a Cox regression model to investigate a possible confounding role of age and gender (*p* = 0.213). Similarly, we compared the survival of patients with mixed ECD-LCH with that of an age- and gender-matched group of isolated ECD (1:2 ratio) selected from the initial cohort and found no significant differences in survival (log-rank *p* = 0.291; Fig. 2).

At univariable Cox regression analysis, the risk of death increased with age at diagnosis (HR 1.079, 95% CI 1.042–1.117; *p* < 0.001), lung involvement (HR 2.974, 95% CI 1.268–6.976; *p* = 0.012), skin papules/patches (HR 3.614, 95% CI 1.522–8.581; *p* = 0.004), associated hematologic conditions (HR 5.890, 95% CI 2.414–14.372; *p* < 0.001), and treatment failure (HR 2.870, 95% CI 1.205–6.836; *p* = 0.017). Conversely, the presence of lytic bone lesions (HR 0.148, 95% CI 0.056–0.387; *p* < 0.001) and pituitary/hypothalamic involvement (HR 0.294, 95% CI 0.121–0.716; *p* = 0.017) showed a negative association with the risk of death.

At multivariable analysis, age at diagnosis (HR 1.052, 95% CI 1.008–1.096), associated hematologic conditions (HR 3.030, 95% CI 1.040–8.827), and treatment failure (HR 9.736, 95% CI 2.919–32.481) predicted an increased risk of death, while the presence of lytic bone lesions was associated with a lower risk (HR 0.116, 95% CI 0.031–0.432) (Table 2).

**Table 2:** Univariable and multivariable Cox regression models investigating predictors of death in patients with mixed ECD-LCH.

	Univariable		Multivariable	
	HR (95% CI)	*p*-value	HR (95% CI)	*p*-value
Age at diagnosis	1.079 (1.042–1.117)	**<0.001**	1.052 (1.008–1.096)	0.019
Female sex	0.685 (0.364–0.685)	0.364		
Organ involvement				
Long bone	0.675 (0.157–2.907)	0.598		
Sclerotic	0.991 (0.292–3.359)	0.988		
Lytic	0.148 (0.056–0.387)	**<0.001**	0.116 (0.031–0.432)	0.001
CNS	0.478 (0.207–1.106)	0.085		
Neurodegenerative disease	0.449 (0.165–1.221)	0.117		
Facial/orbit	0.636 (0.284–1.424)	0.272		
Heart	1.372 (0.598–3.149)	0.455		
Large vessel	1.135 (0.465–2.774)	0.781		
Lung	2.974 (1.268–6.976)	**0.012**		
Interstitial/pleural	1.647 (0.731–3.712)	0.229		
Upper lobes, cystic	3.179 (1.198–8.435)	**0.020**		
Retroperitoneal	2.129 (0.791–5.729)	0.135		
Hypothalamic/Pituitary	0.294 (0.121–0.716)	**0.007**		
Skin	2.564 (0.948–6.934)	0.064		
Xanthelasma-like lesions	0.540 (0.214–1.366)	0.193		
Papules/patches	3.614 (1.522–8.581)	**0.004**		
Lymph nodes	1.671 (0.618–4.519)	0.311		
GI	2.701 (0.970–7.521)	0.057		
Associated hematologic neoplasms^a^	5.890 (2.414–14.372)	**<0.001**	3.030 (1.040–8.827)	0.042
Number of involved sites	1.138 (0.923–1.403)	0.225		
≥4 involved sites	3.730 (0.500–27.813)	0.199		
*BRAF*^*V600E*^ mutation	1.131 (0.421–3.038)	0.807		
Treatment				
BRAFi and/or MEKi (first line)	1.963 (0.742–5.189)	0.174		
BRAFi and/or MEKi (any time)	0.635 (0.282–1.431)	0.273		
Lack of response				
To first-line therapy	0.772 (0.330–1.804)	0.550		
To at least two lines of therapy	0.826 (0.335–2.037)	0.678		
To all lines of therapy	2.870 (1.205–6.836)	**0.017**	9.736 (2.919–32.481)	<0.001

Statistically significant *p*-values are reported in bold.

HR, hazard ratio; 95% CI, 95% confidence interval; CNS, central nervous system; GI, gastrointestinal; BRAFi, BRAF inhibitors; MEKi, MEK inhibitors.

a Including myeloproliferative neoplasm, myelodysplastic syndromes, and non-Hodgkin lymphoma.

## Discussion

In this study, we screened the largest cohort of ECD patients reported so far (502 cases) and identified 69 patients with mixed ECD-LCH (14%). The clinical presentation and long-term outcome of patients with mixed forms were compared with those of patients with isolated ECD. The phenotypes of the two groups were significantly different and patients with ECD-LCH showed a more disseminated disease. The *BRAF*^*V600E*^ mutation was more frequent among patients with ECD-LCH, who also proved amenable to targeted treatments. The long-term outcome of mixed ECD-LCH was comparable to that of ECD alone. However, despite the efficacy of newer therapies, mortality remained remarkable (35% after a median follow-up of 71 months in the mixed group). Our findings are likely to be widely generalizable since the study recruited a large cohort of patients in national referral centers.

The histiocytoses tend to be considered a heterogeneous group of unique conditions. However, patients with histiocytosis frequently show overlap between different forms, and this association is more frequent for ECD and LCH. While LCH typically affects children and ECD adults, the two conditions share multiple clinical features^11^ and pathogenic mechanisms. Indeed, Milne et al. mapped the neoplastic clones of LCH and ECD through *BRAF*^*V600E*^ and detected this mutation in bone marrow and circulating CD34+ stem cells and in blood myeloid cells.^29^ This observation led to the hypothesis that both conditions originate from a common progenitor and can generate different phenotypes that may partially overlap. This concept is also supported by the presence of lesions shared by LCH and ECD such as neurodegeneration, which is supposed to originate from myeloid progenitors through a BRAF-induced senescence program.^30^ Also, LCH and ECD pathology features may coexist within the same tissue biopsy,^11^ Interestingly, histopathological observations suggest an evolution of LCH lytic bone lesions, which can lose LCH immunostaining markers and acquire those typical of ECD, a process usually referred to as xanthomization (Emile JF, in preparation). Altogether, these findings suggest a complexity of histiocytic disorders that goes beyond traditional classifications. New paradigms to describe these conditions are likely to include molecular drivers and consider in a broader perspective the pathomechanisms of disease development.

**Fig. 3:**
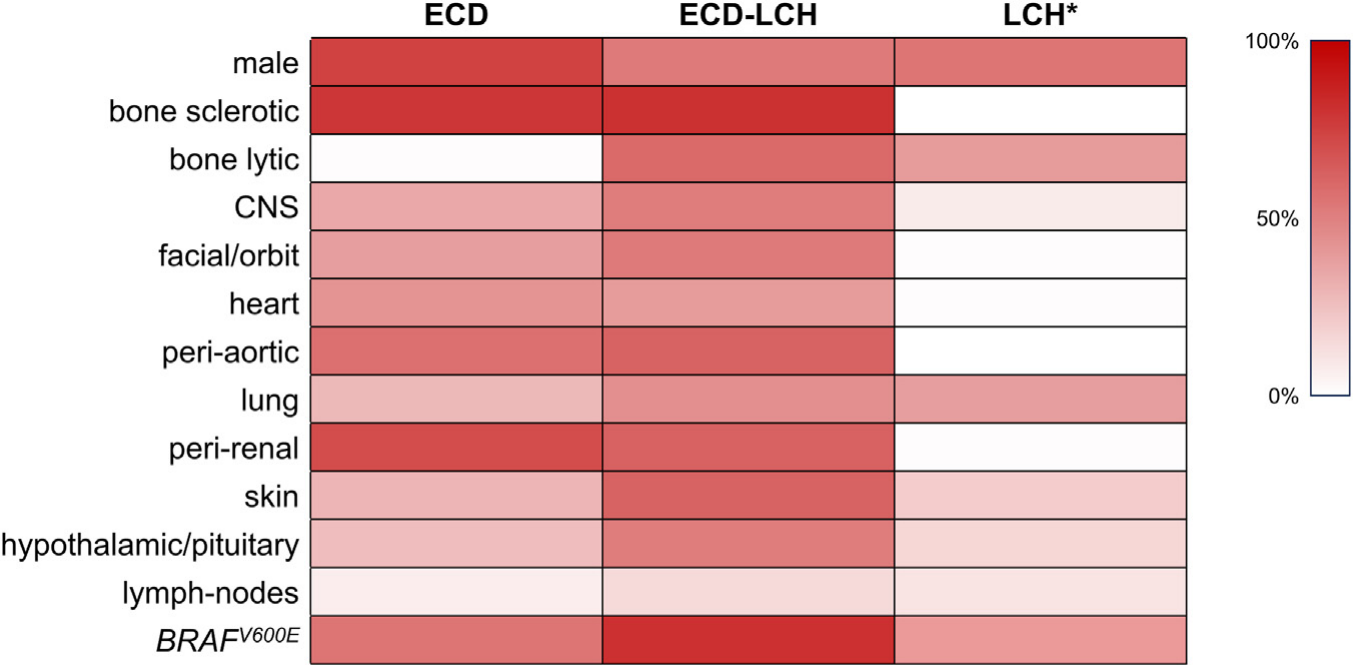
Heat-map of clinical characteristics of patients with ECD, mixed ECD-LCH, and LCH. *Abbreviations used in the figure*: ECD, Erdheim-Chester Disease; LCH, Langerhans Cell Histiocytosis; CNS, central nervous system. ∗Data on patients with LCH are extracted from two recent cohort studies on LCH (DOIs: 10.1111/cas.15879; 10.1182/bloodadvances.2023010706).

In our study, following stringent criteria that required a biopsy-proven LCH diagnosis, the clinical overlap between ECD and LCH was found in 14% of ECD patients. In a previous series of 23 patients, Hervier et al. observed that the clinical spectrum of mixed ECD-LCH was much more reminiscent of that of ECD, compared to LCH,^11^ and our findings are consistent with this observation (Fig. 3). However, while Hervier et al. only showed a lower prevalence of cardiac infiltration and a higher prevalence of hypothalamic/pituitary involvement, we detected several additional differences between the two groups, namely the higher frequency of female individuals and of long bone, CNS, facial/orbit, lung, skin, and lymph node involvement in the mixed group. Patients with mixed phenotypes also had a more disseminated disease and the *BRAF*^*V600E*^ mutation was identified in most of them (81%). Most of these phenotypic differences were confirmed in an age- and gender-matched comparison between mixed forms and ECD alone. Milne and colleagues suggested that ECD and LCH originate from common progenitors that acquire a different phenotype based on the differentiation pattern and the local environment.^29^ Based on this hypothesis, the differences between mixed forms and isolated ECD might reside on the acquisition of the somatic drivers (*BRAF*^*V600E*^) at an earlier stage of myeloid differentiation, which could prime the clonal population to potentially generate both types of lesions.

In around 40% of patients, the LCH diagnosis preceded that of ECD, which highlights the necessity to consider the histiocytoses as a dynamic disease continuum and to carefully screen patients for overlapping involvement both at diagnosis and throughout their follow-up. This consideration particularly applies to patients with an initial LCH phenotype, considering their potential to develop ECD-like lesions even many years after LCH diagnosis.

We analyzed the response to treatment in our cohort focusing on targeted molecules, whose role had not yet been analyzed in mixed ECD-LCH. BRAFi and/or MEKi were able to induce a response in 75% of cases and were more effective than conventional treatments. Also, the use of BRAFi and/or MEKi was negatively associated with treatment failure at univariable analysis, and treatment failure was a strong predictor of death. As many patients with LCH still receive conventional chemotherapy, which might be effective in the context of isolated LCH, this strategy should be avoided if a diagnosis of mixed ECD-LCH is made, considering the poor response rate in these patients. The same holds true for interferon-α (the traditional treatment for ECD), whose efficacy in mixed forms is around 30%. Conversely, BRAFi and MEKi induced sustained responses in most patients. Also considering the limited efficacy of conventional treatments (mostly interferon-α) in the historic cohort,^11^ this study reinforces the indication to use targeted treatments either as first-line or after the development of ECD lesions in patients with a metachronous diagnosis.

Twenty-four patients (35%) died after a median follow-up of 71 months, in most cases due to disease progression or associated neoplasms. The survival analysis showed a similar profile between ECD-LCH and ECD alone. Within the mixed ECD-LCH cohort, the main independent predictors of poor survival included older age at diagnosis, associated hematologic conditions, and treatment failure, while the presence of lytic bone lesions portended a good prognosis. Therefore, it is likely that a continuum exists within this mixed phenotype, with phenotypes more reminiscent of LCH (*e.g.*, lytic lesions, younger age) showing a better outcome than the ECD-skewed ones.

The study has limitations, which include a non-standardized assessment of organ involvement and associated diseases (*e.g.*, hematologic malignancies) that can derive from the long observation period and the different practices between the two centers. Moreover, due to the rarity of the disease, we did not define a sample size for the study. The molecular characterization and the treatment approaches were also non-standardized, although they generally followed the international guidelines, to which our groups contributed. Moreover, our mixed and ECD cohorts were not compared with an adult-onset LCH cohort, which could be of interest and possibly the subject of future research.

To conclude, as compared to ECD alone, mixed ECD-LCH is more frequently a multisystem disease with predominant long bone, CNS, facial/orbit, lung, skin, and hypothalamic/pituitary involvement. Mixed ECD-LCH is driven in most cases by the *BRAF*^*V600E*^ mutation, and patients are sensitive to targeted treatments. Clinical features such as the type of bone involvement, the presence of associated neoplasms, age, and the response to treatment are the main predictors of outcome.

## Contributors

FPeg, AV, and JH conceived the work and wrote the manuscript. FPeg, MP, FC-A, FPey, GL, IT, GB, MdM, TLS, SBa, EO, NF-M, OH, BF, DK, NS, NA, BT, FL, JR, YA, JK, SBu, BH, ATo, AI, ATa, ES, ZA, AV, and JH followed the patients and contributed to data collection. FPey performed the statistical analyses. AB, FC, and JFE performed pathology and molecular studies. QM, SBo, and NS performed radiology studies. All authors had full access to all the data in the study and had final responsibility for the decision to submit for publication. Finally, all authors critically revised the manuscript.

## Data sharing statement

Aggregated data will be made available upon appropriate request after the study publication.

## Declaration of interests

AI reports research grants from Transgene, Sanofi, and Nutritheragene; consulting fees from Novocure, LeoPharma, Polytone Laser, and Novartis; honoraria from Novocure and Neurologies; travel funding from LeoPharma, Novocure, and Carthera.

BT report consulting fees and honoraria from GSK, AstraZeneca, CSL Vifor, Boehringer Ingelheim, and Novartis; advisory board activity for Amgen.

## Appendix ASupplementary data

Supplementary Tables S1–S4

Supplementary Fig. S1 Supplementary Fig. S1. Clinical characteristics at diagnosis of patients with mixed ECD-LCH. *Abbreviations used in the figure*: ECD, Erdheim-Chester Disease; LCH, Langerhans Cell Histiocytosis; CNS, central nervous system; NA, not available.
