# Supplementary material for: Long-term outcome and prognosis of mixed histiocytosis (Erdheim-Chester disease and Langerhans Cell Histiocytosis)
Source: eClinicalMedicine. 2024 May 27;73:102658. doi: 10.1016/j.eclinm.2024.102658 (PMC11152896; doi:10.1016/j.eclinm.2024.102658)
Supplement: Supplementary Tables S1–S4 [file mmc1.docx]

**Table S1.** Biopsy sites

|  | **ECD** | **LCH** |
| --- | --- | --- |
| Bone, n (%) | 15 (25%) | 33 (48%) |
| Skin, n (%) | 12 (20%) | 24 (35%) |
| CNS, n (%) | 3 (5%) | 0 (0%) |
| Facial/orbit, n (%) | 3 (5%) | 1 (1%) |
| Heart, n (%) | 1 (2%) | 0 (0%) |
| Lung, n (%) | 1 (2%) | 5 (7%) |
| Perirenal, n (%) | 24 (41%) | 0 (0%) |
| Lymph nodes, n (%) | 0 (0%) | 3 (4%) |
| Pituitary, n (%) | 0 (0%) | 1 (1%) |
| Liver, n (%) | 0 (0%) | 1 (1%) |
| Peritoneum, n (%) | 0 (0%) | 1 (1%) |
| **Total, n** | 59 | 69 |

ECD, Erdheim-Chester disease; LCH, Langerhans Cell Histiocytosis; CNS: central nervous system

**Table S2.** Differences in baseline clinical characteristics between patients with mixed ECD-LCH and patients with ECD alone matched 1:2 for age and gender.

|  | **Mixed ECD-LCH (N=69)** | **ECD alone**  **(N=138)** | ***p*-value** |
| --- | --- | --- | --- |
| Organ involvement  Long bone  Sclerotic  CNS  Neurodegenerative disease  Facial/orbit  Heart  Large vessel  Lung  Interstitial/pleural  Retroperitoneal  Hypothalamic/Pituitary  Skin  Lymph nodes  GI  Associated hematologic neoplasms*  Number of involved sites  >4 involved sites | 63 (91%)  56 (81%)  35 (51%)  21 (30%)  36 (52%)  27 (39%)  42 (61%)  30 (43%)  21 (30%)  43 (62%)  35 (51%)  42 (61%)  10 (15%)  9 (13%)  11 (16%)  6 (4-7)  56 (81%) | 113 (82%)  113 (82%)  46 (33%)  22 (16%)  56 (41%)  57 (41%)  81 (59%)  39 (28%)  39 (28%)  84 (61%)  42 (30%)  47 (34%)  9 (7%)  12 (9%)  17 (12%)  4 (3-5)  93 (67%) | 0·073  0·898  **0·016**  **0·016**  0·113  0·763  0·956  **0·028**  NA  0·998  **0·004**  **<0·001**  0·611  0·328  0·472  **<0·001**  **0·037** |
| Somatic mutations^#^  *BRAF^V600E^*  *MAP2K1*  No mutations  Other mutation | 54/67 (81%)  5/67 (7%)  8/67 (12%)  0 (0%) | 77/129 (60%)  14/129 (11%)  36/129 (28%)  2/129 (2%) | **0·003**  0·666  **0·011**  NA |

Data are n (%), median (IQR), or n/N (%)

CNS, central nervous system; GI, gastrointestinal; ECD, Erdheim-Chester disease; LCH, Langerhans Cell Histiocytosis; NA, not applicable

^#^ Data not available for all patients

* including myeloproliferative neoplasm, myelodysplastic syndromes, and non-Hodgkin lymphoma

**Table S3.** Treatment and response

| **Class of drug** | **1^st^ line (n= 67)** | | **2^nd^ line (n= 41)** | | **3^rd^ line (n= 16)** | |
| --- | --- | --- | --- | --- | --- | --- |
|  | **n (%)** | **response (%)** | **n (%)** | **response (%)** | **n (%)** | **response (%)** |
| IFNa | 25 (37%) | 8 (32%) | 4 (10%) | 1 (25%) | 3 (19%) | 1 (33%) |
| BRAFi | 13 (19%) | 9 (75%) | 15 (37%) | 11 (73%) | 5 (31%) | 4 (80%) |
| MEKi | 6 (9%) | 5 (83%) | 5 (12%) | 4 (80%) | 4 (25%) | 2 (50%) |
| BRAFi+MEKi | 3 (5%) | 3 (100%) | 4 (10%) | 3 (75%) | 2 (13%) | 2 (100%) |
| Chemotherapy* | 17 (25%) | 5 (29%) | 2 (5%) | 0 (0%) | 0 (0%) | NA |
| Anti-IL1 | 2 (3%) | 0 (0%) | 4 (10%) | 0 (0%) | 1 (6%) | 0 (0%) |
| Anti-TNF | 0 (0%) | NA | 5 (12%) | 1 (20%) | 0 (0%) | NA |
| Others | 1 (2%) | 0 (0%) | 2 (5%) | 0 (0%) | 1 (6%) | 0 (0%) |

IFNa, interferon-alpha; BRAF inhibitors, MEKi: MEK inhibitors; IL1, interleukin-1; TNF, tumor necrosis factor

*chemotherapy regimens included LCH-based protocols (vinblastine and prednisone) in 13 cases, cladribine in four, and methotrexate in two.

**Table S4.** Univariable and multivariable Cox regression analysis investigating predictors of treatment failure in patients with mixed ECD-LCH

|  | **Univariable** | | **Multivariable** | |
| --- | --- | --- | --- | --- |
|  | **OR (95%CI)** | ***p*-value** | **OR (95%CI)** | ***p*-value** |
| Age at diagnosis | 1·014 (0·982-1·046) | 0·396 |  |  |
| Female sex | 0·889 (0·289-2·734) | 0·837 |  |  |
| Organ involvement  Long bone  Sclerotic  Lytic  CNS  Neurodegenerative disease  Facial/orbit  Heart  Large vessel  Lung  Interstitial/pleural  Upper lobes, cystic  Retroperitoneal  Hypothalamic/Pituitary  Skin  Xanthelasma-like lesions  Papules/patches  Lymph nodes  GI  Associated hematologic neoplasms*  Number of involved sites  >4 involved sites | 0·271 (0·049-1·503)  0·265 (0·073-0·962)  1·540 (0·466-5·088)  0·691 (0·223-2·141)  0·646 (0·179-2·329)  0·821 (0·267-2·529)  0·649 (0·197-2·146)  1·075 (0·338-3·422)  1·993 (0·640-6·209)  1·586 (0·485-5·183)  1·731 (0·380-7·891)  0·545 (0·175-1·698)  0·962 (0·313-2·957)  0·763 (0·244-2·385)  0·909 (0·272-3·039)  0·649 (0·197-2·146)  3·067 (0·712-13·208)  0·358 (0·041-3·108)  0·311 (0·036-2·667)  0·885 (0·665-1·179)  1·057 (0·252-4·430) | 0·135  **0·043**  0·479  0·522  0·505  0·732  0·479  0·902  0·234  0·445  0·479  0·296  0·945  0·642  0·877  0·479  0·133  0·352  0·287  0·404  0·940 | 0·295 (0·072-1·204) | 0·089 |
| *BRAF^V600E^* mutation | 0·239 (0·069-0·829) | **0·024** | 0·472 (0·114-1·952) | 0·300 |
| Treatment  BRAFi and/or MEKi (first line)  BRAFi and/or MEKi (any time) | 0·423 (0·106-1·683)  0·308 (0·095-0·996) | 0·222  **0·049** | 0·375 (0·097-1·453) | 0·156 |

OR, odds ratio; 95%CI, 95% confidence interval; CNS, central nervous system; GI, gastrointestinal; BRAFi, BRAF inhibitors; MEKi, MEK inhibitors

* including myeloproliferative neoplasm, myelodysplastic syndromes, and non-Hodgkin lymphoma
