# Supplementary figures and images for: Long-term outcome and prognosis of mixed histiocytosis (Erdheim-Chester disease and Langerhans Cell Histiocytosis)
Source: eClinicalMedicine. 2024 May 27;73:102658. doi: 10.1016/j.eclinm.2024.102658 (PMC11152896; doi:10.1016/j.eclinm.2024.102658)

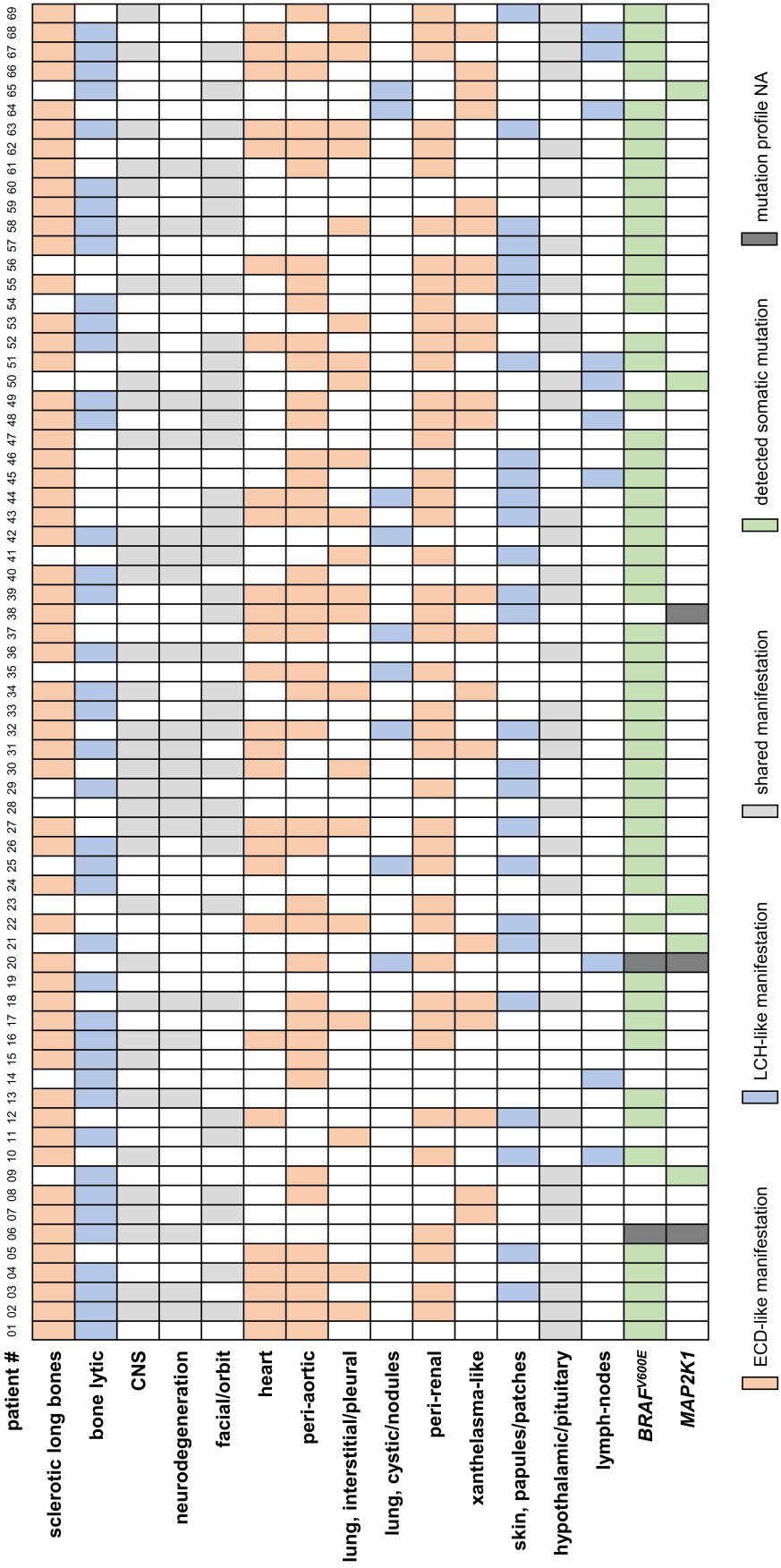

Supplement: Supplementary Fig. S1 — Clinical characteristics at diagnosis of patients with mixed ECD-LCH. Abbreviations used in the figure: ECD, Erdheim-Chester Disease; LCH, Langerhans Cell Histiocytosis; CNS, central nervous system; NA, not available. [file mmc2.pdf]
